# Administering Parenteral Medications in Managing Patients with Acute Arousal in the Behavioral Assessment Unit of the Emergency Department in Hospital Settings

**DOI:** 10.3390/clinpract15060112

**Published:** 2025-06-16

**Authors:** Harshini M. Liyanage, Katy Boyce, Yiting Gong, Theresa Koo, Soumitra Das, Naveen Thomas

**Affiliations:** 1 Western Health, Melbourne 3021, Australia; katy.boyce@wh.org.au (K.B.); theresa.koo@wh.org.au (T.K.); soumitra.das@wh.org.au (S.D.); naveen.thomas@wh.org.au (N.T.); 2 The Faculty of Medicine, Dentistry, and Health Sciences, University of Melbourne, Melbourne 3010, Australia; yitingg@student.unimelb.edu.au

**Keywords:** acute arousal, parenteral medications, emergency department

## Abstract

**Background/Objectives**: The administration of parenteral medications is essential in managing acute arousal within the Behavioral Assessment Unit (BAU) of the emergency department (ED), where timely and effective intervention is critical. This study aims to evaluate current practices surrounding the use of parenteral medications for patients with acute agitation, focusing on adherence to protocols, medication safety, documentation accuracy, and patient outcomes. **Methods**: A retrospective analysis was conducted on 177 cases from December 2023 to February 2024. The study assessed the demographics, diagnoses, treatment protocols, and patient outcomes, with a particular emphasis on the use of parenteral medications such as benzodiazepines and antipsychotics. The relationship between medication administration and involuntary admission, mechanical restraint usage, and patient outcomes was also explored. **Results**: The majority of patients were aged between 21 and 30 years, and there was a predominance of male patients across both groups. Schizophrenia was the most common diagnosis, with a higher prevalence in the parenteral group (34%) compared to the oral-only group (24%), and personality disorders were more frequent in the parenteral group. Intramuscular (IM) medication administration was strongly associated with the use of mechanical restraint, with patients receiving IM medication being 35 times more likely to require restraint, emphasizing the link between more intensive treatment approaches and behavioral challenges. The most frequently administered medications were diazepam (40.6%) and olanzapine (36.5%), with olanzapine, droperidol, and diazepam most commonly used parenterally. Documentation of physical assessments prior to parenteral administration was present in most cases, though comprehensive evaluations such as ECGs were inconsistently performed. **Conclusions**: Parenteral medications, including benzodiazepines and antipsychotics, were effective in rapidly stabilizing patients, but the study emphasizes reducing dependency on mechanical restraints. Tailoring treatment to patient characteristics and employing alternative de-escalation strategies can improve safety and align with recovery-oriented care. This study highlights the need for evidence-based practices to optimize care and improve patient outcomes in ED settings. Further research is needed to explore long-term outcomes and refine non-coercive care approaches.

## 1. Introduction

Acute arousal in psychiatry refers to a heightened state of physiological and psychological activation, often manifesting as agitation, aggression, or severe anxiety [1]. This state is characterized by increased sympathetic nervous system activity, elevated levels of excitatory neurotransmitters such as dopamine and norepinephrine, and decreased inhibitory neurotransmitters like GABA [2]. Such neurochemical imbalances can lead to impaired judgment, increased risk of self-harm or harm to others, and challenges in patient management within emergency settings [1]. In emergency psychiatric settings, the management of acute arousal is critical to ensure patient safety and facilitate timely intervention.

Administering parenteral medications in the management of patients experiencing acute arousal within the Behavioral Assessment Unit of the emergency department represents a critical aspect of hospital care. In this high-stakes environment, where swift and effective intervention is paramount, the accurate administration of medications through parenteral routes is indispensable. This article explores the intricacies and challenges faced by healthcare professionals in this specialized setting, emphasizing the importance of precise assessment, careful medication selection, and vigilant monitoring to ensure optimal patient outcomes. Through a comprehensive examination of protocols, best practices, and the latest research, this article aims to provide insights into the complexities of treating acute arousal in the emergency department, highlighting the pivotal role of parenteral medications in achieving therapeutic success.

Containment measures are used in mental healthcare to manage the risk of harm to patients and staff [3]. Furthermore, the use of these measures raises ethical issues related to patient rights and dignity and runs contrary to recovery-oriented mental healthcare [4]. Rates of administration, type of medication, duration, and mechanical restraint afterward in emergency departments vary widely across countries and between units in the same hospital. These variations can be accounted for by characteristics of the settings and case mix, as well as different definitions and data collection techniques [5,6,7].

Administering parenteral medications to manage acute arousal in patients within the emergency department (ED) of a hospital setting is supported by a growing body of evidence that emphasizes the importance of timely and appropriate pharmacological intervention [8]. Acute arousal, which can present as agitation, aggression, or severe anxiety, poses significant challenges to patient safety and requires swift intervention to stabilize the patient and facilitate further assessment and treatment. Many hospitals and healthcare organizations have developed clinical guidelines and protocols specifically for managing acute arousal in the ED. These guidelines typically recommend the use of parenteral medications in cases where non-pharmacological interventions are insufficient or impractical due to the severity of the agitation or aggression.

Western Health Victoria Guideline for managing patients with acute arousal provides comprehensive guidance regarding this (Western Health Pharmacological Management of Acute Behavioral Disturbance in the Emergency Department Guideline—Supplementary Materials). It is tailored to the needs of the consumer considering risks and past history. Although the Behavioral Assessment Unit employs both pharmacological and non-pharmacological strategies in the management of acute behavioral disturbances, this audit focuses primarily on pharmacological interventions, specifically comparing oral and parenteral medication use. Non-pharmacological approaches—such as verbal de-escalation, trauma-informed care, and sensory modulation—are critical first-line strategies in psychiatric emergencies; however, they were not systematically analyzed in this study due to limitations in retrospective documentation. Their brief mention in the Discussion section serves to contextualize pharmacological use within a broader framework of best practice care and highlight areas for improvement in integrated, non-coercive intervention strategies. Future audits should aim to include these components for a more holistic evaluation.

Studies have demonstrated the safety and efficacy of specific medications, such as benzodiazepines (e.g., lorazepam, midazolam) and antipsychotics (e.g., haloperidol, olanzapine), in rapidly controlling acute agitation and aggression. These medications are administered via parenteral routes (e.g., intramuscular, intravenous) to achieve rapid onset of action and reliable absorption, which is crucial in emergency situations [9]. A systematic review and meta-analysis using 53 papers concluded that olanzapine or haloperidol plus promethazine or droperidol are most effective and safe for use as rapid tranquilization [9]. Midazolam sedates most quickly. However, due to increased saturation problems, midazolam is restricted to use within the emergency department of a general hospital [9].

A study conducted in Turkey found that atypical antipsychotics were as effective as the classic ones and more advantageous in many aspects [10]. The same study concluded that atypical antipsychotics such as risperidone, ziprasidone, and olanzapine with or without benzodiazepines should be considered first in the treatment of acute agitation. If these agents are not available, the combination of a classic antipsychotic and a benzodiazepine would be a reasonable alternative. An oral treatment should always be offered first to build up an alliance with the patient and suggest an internal rather than external locus of control [10].

Evidence suggests that the timely administration of parenteral medications in the ED can lead to improved patient outcomes, including reduced duration of agitation, decreased risk of harm to self and others, and improved compliance with further diagnostic and therapeutic interventions [11]. In a systematic review to assess the effectiveness and safety of intravenous medications for the management of acute disturbances, the results showed mixed but positive results for the effectiveness of IV dexmedetomidine, lorazepam, droperidol, and olanzapine. Evidence was more limited for IV haloperidol, ketamine, midazolam, and valproate [12].

The evidence base also addresses risk management strategies associated with the use of parenteral medications, such as monitoring for adverse effects (e.g., respiratory depression, hypotension) and ensuring appropriate dosing based on patient characteristics (e.g., age, comorbidities) [13]. Effective management of acute arousal in the ED often involves a multidisciplinary approach, where pharmacological interventions are complemented by behavioral strategies, environmental modifications, and collaboration with psychiatric or behavioral health specialists [14].

### Objectives

This retrospective study aimed to assess current practices and protocols surrounding the administration of parenteral medications in the BAU, focusing on adherence to guidelines, medication safety, documentation accuracy, and overall patient outcomes. By evaluating these key aspects, the study aimed to identify areas of strength and opportunities for improvement in the management of acute arousal episodes in the ED setting.

1. To describe the clinical and demographic characteristics of patients receiving oral versus parenteral psychotropic medications in an emergency mental health setting;
2. To explore associations between parenteral medication use and the application of mechanical restraint;
3. To examine prescribing patterns and alignment from guideline-recommended pharmacological management in the Behavioral Assessment Unit (BAU);
4. To identify potential gaps in current practice that could inform future quality improvement initiatives, particularly regarding non-pharmacological interventions and safety monitoring.

## 2. Materials and Methods

### 2.1. Study Design and Setting

This was a retrospective observational study conducted at the Emergency Mental Health Unit of Sunshine Hospital Melbourne Victoria, focusing on patients who required pharmacological intervention or mechanical restraint due to acute behavioral arousal. The study period spanned from 1 December 2023 to 29 February 2024.

### 2.2. Data Source and Extraction

Data were extracted from the hospital’s Electronic Medical Record (EMR) system. Patients who received medication or mechanical restraint for acute arousal were identified through a review of the unit’s handover documents. These documents, maintained daily by the Emergency Mental Health Unit staff, include summary notes of incidents involving behavioral disturbances, interventions applied, and clinical outcomes.

A list of potentially eligible patients was generated based on mentions of acute arousal episodes, use of oral, intramuscular or intravenous sedatives, or application of mechanical restraints. Patient identifiers were replaced with anonymized codes at the time of data extraction to maintain confidentiality.

### 2.3. Inclusion and Exclusion Criteria

Inclusion Criteria:

- Patients presenting with acute behavioral arousal requiring medication or mechanical restraint;
- Seen at the Emergency Mental Health Unit between 1 December 2023 and 29 February 2024.

Exclusion Criteria:

- Cases without adequate documentation in the EMR;
- Patients who were sedated or restrained for non-behavioral medical indications (e.g., seizure, delirium unrelated to psychiatric cause).

### 2.4. Data Analysis

Data were analyzed using R statistical software (version 4.3.3). Descriptive statistics were computed for all variables. Continuous variables such as age and time-to-intervention were summarized using means and standard deviations or medians and interquartile ranges (IQRs), depending on data distribution. Categorical variables (e.g., type of medication used, gender, type of restraint) were summarized using frequencies and percentages.

Comparative analyses were planned where appropriate.

All *p*-values < 0.05 were considered statistically significant.

### 2.5. Ethical Statement

This study was conducted in accordance with the Declaration of Helsinki and approved by the Ethics Committee of Western Health (Project ID 107132, Local Reference Number QA 2024.35) on 10 May 2024. 

## 3. Results

Out of a total of 177 participants, 133 received only oral medication, while 44 received parenteral medication. The majority of the patients were aged between 21 and 30 years. There were more male patients than female patients across both groups. Patients who received parenteral medication were more likely to be involuntary admissions (70%). Schizophrenia was the most common diagnosis overall (27%), with a higher prevalence in the parenteral group (34%) compared to the oral-only group (24%). Substance-induced psychosis was the second most common diagnosis (19%), with similar rates observed in both groups. Personality disorders were more frequent in the parenteral group (14%) compared to the oral-only group (9%) (Table 1).

Notably, personality disorders emerged as one of the more frequent diagnoses among patients receiving parenteral medications. (Table 1) This finding is clinically significant given that there are currently no pharmacological treatments formally approved for the management of personality disorders. The use of antipsychotics and benzodiazepines in these cases likely reflects a need to manage acute behavioral disturbances rather than to treat the underlying disorder. This raises important questions about the appropriateness of pharmacological interventions in such presentations and underscores the importance of developing and implementing alternative, non-pharmacological approaches tailored to the specific needs of this patient population.

Intramuscular (IM) medication administration was significantly associated with the use of mechanical restraint (Table 2). The odds ratio for IM medication was 35.20 (95% CI: 11.5 to 138; *p* < 0.001), indicating that patients who received IM medication were 35 times more likely to require mechanical restraint compared to those who did not. No significant associations were found for involuntary admission (OR = 1.11, 95% CI: 0.36 to 3.50; *p* = 0.854), age, or gender (OR = 1.86, 95% CI: 0.64 to 5.75; *p* = 0.263).

Overall, the most commonly administered medications were diazepam (40.6%) and olanzapine (36.5%). (Table 3) Oral administration was primarily dominated by diazepam (33.0%) and olanzapine (26.7%). For parenteral administration, the most frequently used medications were olanzapine (9.8%), droperidol (9.5%), and diazepam (7.6%). Midazolam, haloperidol, and promethazine each accounted for less than 3% of total administrations. Notably, Acuphase was not used at all during the study period.

## 4. Discussion

This study analyzed the characteristics of patients receiving oral-only versus parenteral (primarily intramuscular) medication and the factors associated with mechanical restraint. The results provide valuable insights into the demographics, clinical profiles, and treatment patterns in psychiatric care.

The Behavioral Assessment Unit (BAU) is a specialized, trauma-informed unit within the emergency department (ED) in Sunshine Hospital, designed to provide comprehensive care for individuals with acute mental health problems. The unit features sensory measures to help patients manage overstimulation, creating a calming environment to support emotional regulation. Staffed with a dedicated clinical team with expertise in trauma and mental health, the BAU includes four beds and ensures 24/7 care with nurses on shift at all times. In addition, security measures are in place to maintain a safe environment for both patients and staff. This unit focuses on delivering immediate, compassionate care while addressing the unique needs of individuals experiencing psychiatric crises.

The primary focus of this study was on pharmacological management due to its central role in the acute stabilization of patients presenting with severe agitation or aggression in emergency mental health settings. In high-acuity environments such as the Behavioral Assessment Unit, rapid symptom control is often necessary to ensure the safety of patients, staff, and others. Parenteral medications, particularly intramuscular antipsychotics and benzodiazepines, are commonly used when patients are unable or unwilling to take oral medication, or when de-escalation efforts are unsuccessful. While this pharmacological focus reflects current clinical practice in acute behavioral emergencies, it is important to acknowledge that medication should not be viewed as the sole or preferred intervention. Evidence-based non-pharmacological strategies, such as verbal de-escalation, trauma-informed engagement, sensory modulation, and structured therapeutic environments, are critical first-line approaches that can prevent escalation and reduce reliance on coercive interventions [15]. Integrating these strategies alongside judicious pharmacologic use can promote more humane, patient-centered care and align with best practice models in emergency psychiatry.

### 4.1. Patient Demographics

The majority of the patients in this cohort were aged between 21 and 30 years (35%), with a similar distribution across both treatment groups. There was no significant difference in age distribution between patients receiving oral-only and parenteral medications. Gender distribution was also similar across groups, with a slight predominance of male patients (51%). These findings suggest that the treatment approach was not influenced by demographic factors such as age or gender.

### 4.2. Diagnosis and Treatment Patterns

The diagnoses listed in the table with a *p*-value of 0.3 indicate no statistically significant difference between patients on oral-only medication and those on parenteral medication. While the *p*-value was greater than the typical threshold of 0.05, suggesting that any observed differences were likely due to random chance, there was a noticeable positive trend in some diagnoses. For instance, schizophrenia and drug-induced psychosis were the most common diagnoses in both groups, though their distribution was not significantly different. Similarly, schizoaffective disorder and personality disorders appeared slightly more frequently in the parenteral medication group, but again, this difference did not reach statistical significance. Notably, despite these variations, it is clear that challenging behaviors can occur across all diagnoses, regardless of the medication group. This emphasizes that the presence of challenging behavior is not exclusive to any particular diagnosis or medication type, highlighting the complexity of psychiatric conditions and their management.

In schizophrenia, substance use disorders are associated with greater positive symptom severity, more suicidal ideation, aggression, and violence [16]. Our study found that substance-induced psychosis was the second most common diagnosis (19%) and showed similar rates across the two groups, indicating that the choice of medication route for this condition may depend on other clinical factors, such as the severity of agitation or cooperation with treatment.

The frequent use of parenteral medications in patients with personality disorders, despite the absence of approved pharmacological treatments for these conditions, highlights a critical gap in current emergency care protocols. This suggests a reliance on symptom-driven pharmacologic interventions in the absence of targeted therapies, reinforcing the urgency of integrating evidence-based, non-pharmacologic strategies such as structured de-escalation techniques and early psychological support into emergency mental healthcare practices.

### 4.3. Physical Examination and Medical Assessment

An important consideration in the emergency administration of parenteral medications—particularly antipsychotics and benzodiazepines—is the medical evaluation undertaken prior to intervention. In the context of our study, baseline physical assessments, including vital signs, brief neurological checks, and evaluation of cardiovascular risk factors, were conducted in most cases prior to administering medications. However, comprehensive assessments such as electrocardiograms (ECGs) were not consistently performed due to the urgency of managing acute behavioral disturbances in the BAU setting.

ECGs are particularly relevant when administering certain antipsychotics known to prolong the QT interval (e.g., haloperidol or ziprasidone), as well as in patients with known cardiac history or concurrent medication use that may affect cardiac conduction. While protocol guidelines in our service recommend ECGs when clinically indicated and when the situation allows, real-world application in acute agitation cases often limits the feasibility of timely ECG acquisition. Patients in severe behavioral distress may not tolerate the procedure, and clinical prioritization is often given to rapid tranquilization and the safety of staff and patients.

The absence of consistent ECG use represents a potential gap in risk mitigation, particularly in cases where high-potency antipsychotics are employed. This highlights the tension between the immediacy of behavioral control and the ideal standard of medical monitoring. In future practice, the integration of rapid point-of-care ECG technologies and structured clinical decision tools may facilitate more consistent cardiac risk screening without impeding the timeliness of care.

Moreover, staff training in the recognition of cardiac risk indicators and the incorporation of risk stratification algorithms into ED protocols may support safer prescribing practices. These enhancements could improve the balance between efficacy and safety in the acute pharmacological management of agitation and should be considered in future protocol revisions and studies.

### 4.4. Voluntary Status and Mechanical Restraints

A significant association was observed between involuntary admission and the use of parenteral medication (*p* = 0.013). Patients receiving parenteral medication were more likely to be involuntarily admitted (70%) compared to those on oral medication (49%). This is consistent with the clinical practice of using parenteral medication to manage acute agitation or aggression, often seen in involuntary admissions.

For seclusion, manual restraint, and mechanical restraint, communication with staff and family during the intervention was a factor preferred by patients, to make the experience of the intervention more acceptable and humane [17]. In our study, parenteral medication was also strongly associated with the use of mechanical restraints. Patients receiving intramuscular (IM) medication were 35 times more likely to require mechanical restraint compared to those receiving oral medication (OR = 35.20, 95% CI: 11.5–138; *p* < 0.001). This underscores the close link between the need for parenteral medication and acute behavioral disturbances that necessitate restraint for the safety of the patient and others. This suggests that patients who are given IM medication might be experiencing conditions that trigger more intense behavioral reactions, making them more prone to needing physical restraint.

The strong association between intramuscular (IM) medication administration and the use of mechanical restraint observed in this study (patients receiving IM medication were 35 times more likely to be restrained) is consistent with the clinical reality that restraint is often used when patients are unable or unwilling to accept oral medication and present with severe agitation or aggression. However, while this correlation is understandable, it underscores the need for a critical evaluation of restraint practices and the development of alternative strategies that prioritize safety while minimizing coercion.

Efforts to avoid physical restraint should begin with early recognition of agitation and the proactive use of non-coercive de-escalation strategies. These include verbal de-escalation techniques, sensory modulation, calming environments, and the early offer of oral medications with patient involvement in decision-making. Training staff in trauma-informed care and early warning signs of escalation can help avoid confrontational situations. Moreover, incorporating peer support workers or mental health clinicians skilled in behavioral intervention into the ED team may improve engagement and reduce the need for restraint.

Mechanical restraint carries well-documented risks, including physical injury (e.g., bruising, fractures); psychological trauma; positional asphyxia; and in rare cases, sudden death—particularly when used in combination with sedative medications. The practice can also severely impact therapeutic rapport and may contribute to post-traumatic stress symptoms, especially in patients with a history of trauma or coercive psychiatric experiences.

In our study setting, mechanical restraint involved the use of soft limb restraints applied by trained staff in accordance with hospital protocol. Restraint was employed only when the patient posed an immediate risk to themselves or others, and less restrictive options were not viable. The initiation of restraint was documented in clinical records, and all cases required authorization and regular review.

Patients who were physically restrained were continuously monitored by nursing staff, including regular assessments of vital signs, circulation, respiration, and mental status. Where possible, monitoring followed national guidelines, which recommend checks at least every 15 min during restraint and continued observation after release. In addition, if parenteral sedation was administered, observations were maintained to detect signs of respiratory depression, oversedation, or paradoxical reactions.

No severe adverse effects directly attributable to parenteral medication administration were documented in the sample. Importantly, no cases of neuroleptic malignant syndrome (NMS), acute dystonia, or other severe extrapyramidal symptoms were reported. However, the retrospective design may limit the capture of all complications, especially if minor effects were underreported. A small number of patients experienced transient hypotension or over-sedation following intramuscular (IM) administration, consistent with known side effects of benzodiazepines and antipsychotics. These were managed supportively and did not result in long-term harm based on available follow-up data. Nonetheless, the combination of physical restraint and sedative medication necessitates careful and consistent monitoring due to the increased risk of respiratory and cardiovascular complications.

Overall, these findings highlight the need to strengthen multidisciplinary protocols that promote early intervention and non-coercive care models and to ensure robust documentation and post-incident review to enhance both patient safety and service quality.

### 4.5. Medication Usage Patterns

The medication usage patterns observed in this audit revealed a preference for diazepam and olanzapine, with diazepam accounting for 40.6% and olanzapine for 36.5% of total administrations. Parenteral administration, which is the core focus of rapid tranquillization as defined by the NICE guidelines [18], was most commonly achieved using olanzapine (9.8%), droperidol (9.5%), and diazepam (7.6%).

However, the infrequent use of lorazepam (2.5% parenterally) and the very limited application of haloperidol combined with promethazine—both of which are first-line agents recommended by NICE—may reflect factors such as local prescribing preferences, cost considerations, availability, and clinician familiarity with certain medications. Diazepam is often preferred over lorazepam by many clinicians due to its longer duration of action. Additionally, limited training and restricted availability of lorazepam during the study period may have influenced its underutilization. These findings highlight the need for the development of local guidelines that are aligned with international best practice recommendations, such as those provided by NICE [19].

The complete absence of Acuphase and the low use of midazolam and haloperidol raise questions about clinicians’ familiarity with or confidence in the guideline-recommended options for managing acute behavioral disturbance. These findings highlight a potential gap in alignment with NICE recommendations, particularly in situations where limited information is available to guide medication choice, or where cardiovascular risk should dictate the use of lorazepam over haloperidol. The reliance on diazepam, despite its less prominent role in current guidelines for rapid tranquillization, may reflect local prescribing culture, medication availability, or clinician preference. Greater adherence to NICE guidelines, alongside structured training and protocol reinforcement, could enhance the safety, consistency, and effectiveness of pharmacological interventions in acute psychiatric emergencies.

### 4.6. Comparison with NICE (National Institute for Health and Care Excellence) Guidelines 2015

The findings of this study, particularly the association between parenteral medication and mechanical restraint, raise important considerations when examined in light of current NICE guidelines (NG10) on the management of violence and aggression in mental health settings [18].

According to NICE Guidelines 2025 [19], mechanical restraint should only be used in high-secure settings or during transfers between medium- and high-secure units, and strictly as a last resort in cases of extreme violence or self-injurious behavior. In the present study, however, mechanical restraint was observed in 38% of patients receiving parenteral medication, which was overwhelmingly administered in response to acute agitation or aggression rather than in high-secure settings. This raises concerns about potential non-compliance with national policy recommendations, suggesting either a broader use of mechanical restraint than is advised or a misclassification of care settings.

The high odds ratio (OR = 35.20, *p* < 0.001) linking parenteral medication to mechanical restraint suggests that these interventions are frequently co-administered. While this may reflect the clinical need for urgent behavioral control, NICE clearly emphasizes that rapid tranquillization—using parenteral medications like IM lorazepam or haloperidol with promethazine—should be prioritized only when oral medications are not feasible [18]. Furthermore, the guidelines caution that each case should be tailored to individual patient factors, including history of response, comorbid conditions, and preferences [18]. The study data do not clarify whether such considerations were systematically addressed, underscoring the need for more rigorous adherence to personalized prescribing practices.

Moreover, the frequent use of pharmacological interventions in patients with personality disorders is noteworthy. NICE guidelines acknowledge the use of medications to manage acute agitation, but pharmacological treatment for personality disorders per se is not generally supported. The relatively high use of parenteral medication in this group (14% vs. 9% in the oral-only group) potentially indicates a gap in access to or implementation of non-pharmacological de-escalation strategies, such as structured psychological support or therapeutic engagement, which NICE advocates as first-line approaches in managing behavioral disturbances.

### 4.7. Comparison with Western Health Guidelines

The pharmacological interventions reported in this study align broadly with Western Health Guidelines for managing aggression, particularly in the stepped approach based on behavioral severity. The data indicate that diazepam (40.6%) and olanzapine (36.5%) were the most commonly used medications, with oral routes being preferred, consistent with guideline recommendations for managing mild agitation and escalating behavior. The guidelines suggest oral diazepam and olanzapine as first-line agents in these phases, aiming to de-escalate behavior and prevent a crisis. In more severe scenarios, such as behavioral crises or emergencies, parenteral administration is typically required. The use of parenteral olanzapine (9.8%) and droperidol (9.5%) supports this, as both are recommended for rapid sedation during acute aggression. However, midazolam and ketamine—highlighted in the guidelines for emergency sedation—were used less frequently (2.2% and 0%, respectively), suggesting possible clinical hesitation or institutional protocols that prioritize other agents. The absence of Acuphase use, despite its mention in broader psychiatric guidelines, may reflect local practice preferences or formulary restrictions. Overall, the medication usage in the study reflects guideline adherence, particularly in early intervention stages, though there may be underutilization of some parenteral agents in behavioral emergencies.

### 4.8. Implications for Clinical Practice

These findings highlight the importance of tailoring treatment strategies based on patient characteristics and clinical needs. The higher prevalence of schizophrenia and personality disorders in the parenteral group suggests that these patients may present with more severe symptoms or challenging behaviors. The strong association between parenteral medication and mechanical restraint calls for careful consideration of alternative de-escalation strategies to minimize the use of restraints wherever possible.

Additionally, the lack of significant associations between parenteral medication use and demographic factors such as age or gender suggests that clinical judgment, rather than demographic characteristics, drives the choice of treatment. However, the high prevalence of involuntary admissions in the parenteral group warrants further exploration into the systemic and clinical factors contributing to this pattern.

Existing research on gender and age in relation to restrictive interventions often suggests that these factors can influence the likelihood of needing restraints, with some studies showing varying rates based on demographic characteristics [20]. However, our study does not align with these findings, as we found that illness severity is the most significant factor in determining the need for restrictive interventions. In cases of severe illness, early intervention, community-based treatment, and mobile support teams are crucial in managing symptoms and reducing the burden on emergency departments.

### 4.9. Limitations and Future Directions

This study offers valuable insights into the use of oral and parenteral medications for behavioral emergencies in an acute psychiatric setting, particularly in relation to mechanical restraint and prescribing patterns. However, several limitations should be considered when interpreting the findings.

The retrospective, cross-sectional design inherently limits the ability to establish causal relationships. Although the study identified associations between parenteral medication use and mechanical restraint, it cannot determine whether the medication directly contributed to the need for restraint or whether both were responses to the same underlying behavioral crisis. Similarly, while demographic and diagnostic trends were described, the design precludes temporal or predictive conclusions.

The quality and consistency of documentation varied across clinical records. Key safety practices—such as baseline physical assessments, electrocardiograms (ECGs) prior to antipsychotic administration, and ongoing monitoring after sedation—were not uniformly recorded. This inconsistency makes it difficult to assess adherence to safety protocols or draw firm conclusions about the quality of risk mitigation. Although no serious adverse events were reported, underdocumentation and retrospective data capture may have led to the omission of minor or delayed complications.

The study also did not explore the clinical decision-making processes that influenced medication route selection, timing of intervention, or the use of mechanical restraint. These decisions are often shaped by complex factors including clinician experience, patient history, and real-time behavioral cues. Without qualitative or observational data to contextualize these factors, it remains challenging to evaluate the appropriateness of interventions or to identify barriers to best practice.

Discrepancies between local prescribing practices and national guideline recommendations (e.g., NICE guidelines) were observed, yet the reasons behind these variations were not examined. Influences such as clinician familiarity, medication availability, local protocols, and resource constraints may have contributed but were not systematically evaluated. Understanding these factors can more deeply inform targeted quality improvement efforts.

Although the study aimed to identify areas for improvement in managing acute behavioral disturbances, it did not assess the impact of specific interventions, training programs, or protocol changes. As a result, while it offers a useful foundation for future service development, it does not propose actionable strategies or evaluate outcomes over time. Prospective studies incorporating standardized outcome measures—such as time to calming, symptom resolution, or restraint duration—alongside structured safety protocols are needed to better understand the effectiveness and safety of interventions in this setting.

## 5. Conclusions

This study highlights key patterns in the use of oral versus parenteral medication for acute behavioral disturbance in a psychiatric emergency setting and quantifies the strong association between intramuscular (IM) administration and mechanical restraint (OR = 35.20, 95% CI: 11.5–138; *p* < 0.001). Such findings reflect real-world clinical decision-making in high-acuity environments, where rapid tranquilization is often prioritized to ensure safety. However, they also raise critical concerns about the potential overreliance on pharmacological and coercive interventions, particularly in patients with diagnoses such as personality disorders, where evidence-based non-pharmacological alternatives are underutilized.

Despite observing no statistically significant differences in demographic or diagnostic profiles between treatment groups, the increased prevalence of involuntary admissions and the use of restraint in the parenteral group suggests that treatment decisions are driven more by illness acuity than by demographic characteristics.

Findings in our study suggest that while the use of parenteral medication aligns with NICE guidance and Western Health Guidelines in acute behavioral crises, the high correlation with mechanical restraint and its possible overuse outside of high-secure environments calls for an urgent review of local practices. This includes ensuring the availability and consistent use of non-pharmacological de-escalation techniques, adherence to legal and ethical standards around restraint, and individualized treatment planning based on NICE’s structured recommendations. However, it is important to recognize that the NICE guidelines may not be fully applicable to our local demographic, where a higher prevalence of dual diagnosis and severe behavioral dysregulation often necessitates more intensive interventions; this underscores the urgent need for context-specific guidelines tailored to the unique clinical and environmental challenges of our setting.

While the analysis revealed no significant differences in diagnosis between the two treatment groups, the strong association between parenteral medication and mechanical restraint highlights the complex interplay between treatment approach and behavioral challenges. This underscores the need for targeted, individualized care strategies, especially for patients with severe symptoms or involuntary admissions, to minimize the use of restrictive interventions. Enhancing clinician awareness, improving access to recommended medications, and reinforcing adherence through targeted training may help bridge the gap between practice and policy. Further research into alternative de-escalation methods and systemic factors influencing the use of parenteral medication and restraints is essential to improving patient care and outcomes in psychiatric settings.

## Figures and Tables

**Table 1:** Participant characteristics (total = 177).

Characteristic	Overall N = 177 ^1^	Patients with Only Oral Medication N = 133 ^1^	Patients with Parenteral Medication N = 44 ^1^	*p*-Value ^2^
Age Group				>0.9
<20	26 (15%)	20 (15%)	6 (14%)	
>60	3 (1.7%)	2 (1.5%)	1 (2.3%)	
21–30	62 (35%)	44 (33%)	18 (41%)	
31–40	44 (25%)	35 (26%)	9 (20%)	
41–50	28 (16%)	21 (16%)	7 (16%)	
51–60	14 (7.9%)	11 (8.3%)	3 (6.8%)	
Gender				>0.9
Female	84 (48%)	63 (48%)	21 (48%)	
Male	90 (51%)	67 (51%)	23 (52%)	
Other	2 (1.1%)	2 (1.5%)	0 (0%)	
Voluntary Status				0.013
Yes	81 (46%)	68 (51%)	13 (30%)	
No	96 (54%)	65 (49%)	31 (70%)	
Diagnosis				0.3
Schizophrenia	47 (27%)	32 (24%)	15 (34%)	
Schizoaffective Disorder	12 (6.8%)	7 (5.3%)	5 (11%)	
Mood Disorders/Affective Disorders	29 (16%)	25 (19%)	4 (9.1%)	
Delusional Disorder	2 (1.1%)	2 (1.5%)	0 (0%)	
Personality Disorders	18 (10%)	12 (9.0%)	6 (14%)	
PTSD	3 (1.7%)	3 (2.3%)	0 (0%)	
Other	18 (10%)	16 (12%)	2 (4.5%)	
Drug-Induced Psychosis	34 (19%)	24 (18%)	10 (23%)	
Psychosis + Personality Disorders	3 (1.7%)	2 (1.5%)	1 (2.3%)	
Affective Disorders + Personality Disorders	8 (4.5%)	8 (6.0%)	0 (0%)	
Anxiety Disorders + Obsessive Compulsive Disorder	3 (1.7%)	2 (1.5%)	1 (2.3%)	
Number of Mechanical Restraints				>0.9
1	10 (38%)	1 (25%)	9 (41%)	
2	5 (19%)	1 (25%)	4 (18%)	
3	0	0	0	
4	11 (42%)	2 (50%)	9 (41%)	

^1^ n (%); ^2^ Fisher’s exact test; Pearson’s Chi-squared test.

**Table 2:** The relationship between parenteral medication administration and mechanical restraint.

Variable	Odds Ratio	95% CI	*p*-Value
Parenteral medication	35.20	(11.5, 138)	<0.001
Involuntary	1.11	(0.36, 3.50)	0.854
Age			
Age > 60	0.00	-	0.993
Age 21–30	0.83	(0.16, 4.49)	0.825
Age 31–40	1.22	(0.21, 7.48)	0.825
Age 41–50	0.70	(0.10, 4.87)	0.713
Age 51–60	0.30	(0.01, 3.76)	0.387
Gender			
Male	1.86	(0.64, 5.75)	0.263

**Table 3:** Frequency of medications used.

Medication	Oral (*n* *)	Oral (%)	Parenteral (*n* *)	Parenteral (%)	Total (*n* *)	Total (%)
Diazepam	104	33.0	24	7.6	128	40.6
Olanzapine	84	26.7	31	9.8	115	36.5
Droperidol	1	0.3	30	9.5	31	9.8
Lorazepam	18	5.7	8	2.5	26	8.3
Midazolam	0	0.0	7	2.2	7	2.2
Haloperidol	0	0.0	4	1.3	4	1.3
Promethazine	0	0.0	4	1.3	4	1.3
Acuphase	0	0.0	0	0.0	0	0.0

* *n* = number.

## Data Availability

No data except the data what is presented in the paper are available.

## Supplementary Materials

The following supporting information can be downloaded at: https://www.mdpi.com/article/10.3390/clinpract15060112/s1.

## Abbreviations

The following abbreviations are used in this manuscript:

BAU	Behavioural Assessment Unit
ED	Emergency department
EMR	Electronic Medical Record
IM	Intramuscular
NMS	Neuroleptic malignant syndrome
NICE	National Institute for Health and Care Excellence
