# Supplementary material for: Administering Parenteral Medications in Managing Patients with Acute Arousal in the Behavioral Assessment Unit of the Emergency Department in Hospital Settings
Source: Clin Pract. 2025 Jun 16;15(6):112. doi: 10.3390/clinpract15060112 (PMC12191530; doi:10.3390/clinpract15060112)
Supplement: Supplementary file 1 [file clinpract-15-00112-s001.zip › clinpract-3600347-supplementary.pdf]

Western Health

Pharmacological Management of Acute Behavioural Disturbance in the Emergency Department Guideline

Document type: Departmental Guideline	Current version: June 2023
Previous version: February 2020	Next review date: June 2026

This document is relevant to all WH sites, including Bacchus Marsh, Melton and Caroline Springs

Contents

1. Overview 2
2. Applicability 2
3. Responsibility 2
4. Authority 2
5. Associated Documentation 2
6. Credentialing Requirements 3
7. Definitions and Abbreviations 3
   7.1 Definitions 3
   7.2 Abbreviations 3
8. Guideline Detail 4
9. Document History 8
10. References 8
11. Sponsor 8
12. Authorisation Authority 8

Appendix 1 (if applicable) 14

1. Overview

This guideline aims to assist in the **initial** pharmacological management of the patient with acute behavioural disturbance in the **Emergency Department**. / **Urgent care Centres.** Pharmacological management should only be employed after exhausting other options. It is not designed to replace clinical judgement. Medical involvement, appropriate investigation and continuous reassessment are required. All management needs to align with the *Medical Treatment Planning and Decisions Act 2016*.

2. Applicability

This guideline applies to all nursing and medical staff involved with the assessment and treatment of agitated patients in the Emergency Departments and Urgent care Centres at Western Health.

3. Responsibility

It is the responsibility of the Nurse Unit Managers and Directors of the Emergency Departments and Urgent care Centres at WH to ensure staff are familiar with this guideline and its content, that it is implemented as outlined within, and that there is compliance by all staff.

4. Authority

Exceptions to the practices described in this guideline can only be authorised by the Directors of the Emergency Departments and Urgent Care Centres.

5. Associated Documentation

In support of this guideline, the following Manuals, Policies, Instructions and/or Guidelines apply:

1. Associated Documentation

In support of this guideline, the following Manuals, Policies, Instructions, Guidelines, or Forms apply:

Code	Name
P-CM3	Medical Consent
P-GC6	Medication use and Management
OG-GC2	Delirium Guidelines
OG-GC6	Alcohol Withdrawal
OP-CC2	Consultation Liaison (CL) Psychiatry and Emergency Mental Health (EMH) Service
OP-CC4	Management of Section 351 Patients in the Emergency Department
OP-CC4	Patient Observation (Specialling)
OP-CM3	Management of Unknown Substances (including Illicit and Unidentifiable Substances)
OP-CM3	Mechanical Restraint, Assessment and Application (Patients)
OP-EP4	Prevention and Management of Occupational Violence and Aggression (OVA)
OP-GC1	Missing Patient and Patient at Risk of Going Missing
OP-GC6	Medical Management of Drug Dependent Persons and Prescription of Long-Term Opioid Medication (including Oncology Patients).
OP-GC6	Medication Prescription, Supply, Storage and Administration
OP-RS5	Contacting Police in Urgent Situations
OP-RS5	Escorting Non-compliant Persons from a Western Health Site
OP-RS5	Management of Code Grey and Duress Alarms
OP-RS5	Management of Suspicious Behaviour
OP-RS5	Managing Requests from Police
OP-RS5	Security Surveillance Management
OP-RS5	Smoke-Free Workplace
OP-RS5	Weapons within Western Health
Emergency Department DP-CM2	Suspected Child Abuse and Neglect in Children
Emergency Department DP-RS5	Behavioural Assessment Room (BAR)
AD351	Mechanical Restraint Observation Record.
SDSO SEC 5.4.5B	Wearable Audio Video Recording System (WAVRS) (local security procedure)
WHAD51.2a	Emergency Department Adult Flow Chart – Behaviours of Concern Observations
	MHA140, 141, 142Mental Health Act, Sections' 111, 112, 114, 115, 116
	Approval/Authority for Restrictive Interventions and Clinical Observations

Appendix 1	Management of Acute Behavioural Disturbance in the Emergency Department - Children <16years or < 50kg
Appendix 2	Management of Acute Behavioural Disturbance in the Emergency Department - Adults < 65 years
Appendix 3	Management of Acute Behavioural Disturbance in the Emergency Department - Older Adults > 65 years
Appendix 4	Behaviours of Concern (BOC) Chart
Appendix 5	HEADSSS Assessment (Psychosocial Screening)

6. Credentialing Requirements

All Emergency Department and Urgent Care staff are required to complete Western Health's "Management of Workplace Violence and Aggression in Healthcare" training program. Staff working in areas classified as 'high' to 'extreme' risk areas are required to complete the one-day initial training course (three core modules) and a 1-hour refresher course every six months.

7. Definitions and Abbreviations

Include here all definitions of terms or abbreviations used in the guideline. It is preferable that pre-existing definitions are used.

7.1 Definitions

For purposes of this guideline, unless otherwise stated, the following definitions shall apply:

Monitored Area	Refers to a resuscitation area in the Emergency Department and UCC where 1:1 nursing, cardiac and respiratory monitoring is possible, and the space allows for security and other staff presence.
Behavioural Assessment Room	'A specifically designed room for assessing and managing emergency department patients exhibiting aggression that places themselves or others (including staff) at risk of harm.' (Guidelines for behavioural assessment rooms in emergency departments DHHS Victoria May 2017)

7.2 Abbreviations

For purposes of this guideline, unless otherwise stated, the following abbreviations shall apply:

4AT	Rapid clinical Test for Delirium
ADHA	Attention Deficit Hyperactivity Disorder
BAR	Behavioural Assessment Room
BMUC	Bacchus Marsh Urgent Care
BOC	Behaviour of Concern
BOCR	Behaviour of Concern Room
BPSD	Behavioural and Psychological Symptoms of Dementia
CAM	Confusion Assessment Method
HEADSSS	Home, Education/Employment, Activities, Drugs, Sex and relationships, Self-harm and depression, Safety and abuse.
IM	Intramuscular
IV	Intravenous
MHUC	Melton Health Urgent care
ODT	Oral Disintegrating tablet
PO	Oral
RCF	Residential Care Facility
SAT	Sedation Assessment Tool
SL	Sublingual
UCC	Urgent Care Centres

8. Guideline Detail

8.1 Children < 16 years or < 50 kg

General Considerations:

- Weight-based dosages.
- Factors include learning, developmental and intellectual disabilities, ADHD, fear and social comorbidities.
- Accidental ingestion as a potential cause.
- Physical size does not equal emotional maturity.

If appropriate, all patients should:

- Be nursed in a quiet area.
- Have continuing verbal de-escalation
- Be provided with food and fluids and toileting access.
- Have the company of family members as appropriate (ideally with consent from both parties).

Before considering pharmacological measures:

- Consider de-escalation: behaviour assessment room (BAR/BOCR), low stimulus area, talking down, timeout, distraction, use of relatives if suitable.
- Address fears, concerns and causation.
- If possible and as able
    - Take a history, check drug/alcohol use, allergies & medications, consider drug interactions & contraindications, and complete the HEADSSS assessment (*Appendix 4*)
    - Do a physical examination, check for inter-current illness and consider alternative diagnoses.
    - Do a mental state assessment.
    - Establish a working diagnosis.

Risk assessment:

- Formal behavioural assessment scales are not part of routine observations for paediatric patients. However, the principles of identifying risk factors based on history and examination, continuous evaluation and early intervention to prevent escalation and distress still apply.

**Patient Behaviour**	**Mild Agitation**<br><br>Anxious and agitated But able to be redirected and reassured	**Escalating Behaviour**<br><br>Becoming more agitated with a possible risk of loss of self-control and aggression	**Behavioural Crisis**<br><br>Severe agitation and distress posing risk to patient and staff safety	**Behavioural Emergency**<br><br>Persisting aggressive behaviour despite sedation
**Aim of Intervention**	Maintain safe environment	Prevent a crisis situation	Restore a safe environment	Maintain staff and patient safety at all times
**Immediate Considerations**	Introduce non-pharmacological strategies, and involve family member(s) if appropriate	Maximise non-pharmacological strategies<br>Consider BAR/BOCR<br>Low stimulus environment	Code grey – Security present<br>Least restrictive practice<br>Consider BAR/BOCR if appropriate	Continued security presence<br>Move to a monitored area<br>ED consultant /senior registrar/ CMO (UCC) involvement
**Medication**	In a child with a known behavioural disorder, consider giving an additional dose of the patients usual medication<br><br>**Olanzapine wafer/ODT SL**<br>< 40 kg 2.5-5 mg SL (max 10 mg daily)<br>> 40 kg 5-10 mg SL (max 20 mg daily)<br><br>OR<br><br>**Diazepam (available in liquid form) PO**<br>0.2-0.4 mg/kg PO<br>Max 10 mg PO		**Droperidol IM**<br>0.1-0.2 mg/kg (max 10 mg)<br><br>OR<br><br>**Olanzapine IM**<br>< 40 kg 5 mg<br>> 40 kg 10 mg<br>Repeat in 15 minutes – if not settling, **escalate to Behavioural Emergency**	**Ketamine IM/IV**<br>4 mg/kg IM<br>(max 400mg)<br><br>OR<br><br>1mg/kg IV<br>(max100mg)<br><br>OR<br><br>**Midazolam IM/IV**<br>0.1-0.2 mg/kg<br>IM or IV<br>(max 20 mg/24 hours)

8.2 Adults < 65 years

General Considerations:

- Drug & alcohol intoxication is common.
- Prescribed anti-psychotic and benzodiazepine medications are common.
- Possible tolerance to medications.
- Consider organic causes/ageing-related diseases in those aged 55-65 – stroke, delirium, dementia, seizures.

Contraindications:

- Head injury.
- Obvious medical cause for agitation e.g. post-ictal, CNS infection.

If appropriate, all patients should:

- Be nursed in a quiet area.
- Have continuing verbal de-escalation.
- Be provided with food and fluids and toileting access.

Before considering pharmacological measures:

- Utilise de-escalation: behavioural assessment room (BAR/BOCR)/single room, low stimulus area, talking down, timeout, distraction, use of relatives if suitable.
- Address fears, concerns and causation.
- If possible and as able:
    - take a history, check drug/alcohol use, allergies, and consider drug interactions and contraindications.
    - do a physical examination, check for inter-current illness and consider alternative diagnoses.
    - do a mental state assessment.
    - establish a working diagnosis.

Risk assessment:

- All patients are subject to the behaviour of concern observations (*Emergency Department Adult Flow Chart – Behaviours of Concern Observations WHAD51.2a*).
- BOC scores > 2 generally require a code grey and pharmacological management.
- BOC scores of 2 and below should not preclude pharmacological management if other risk factors (e.g. history of violence and disruptive behaviour or worrying trends) are present.

**Patient Behaviour**	**Mild Agitation BOC-score 0**<br><br>Anxious and agitated Controlling their behaviour	**Escalating Behaviour BOC-score 1-2**<br><br>Escalation of aggressive behaviour, reduced capacity to control emotions and behaviour	**Behavioural Crisis BOC-score >2**<br><br>Aggressive behaviour is overt, imminent safety threat	**Behavioural Emergency BOC-score >2**<br><br>Persisting aggressive behaviour despite sedation
**Aim of Intervention**	Maintain safe environment	Prevent a crisis situation	Restore a safe environment	Maintain staff and patient safety at all times
**Immediate Considerations**	Consider BAR/BOCR<br>Low stimulus environment	Consider BAR/BOCR<br>Low stimulus environment	Code grey<br>Consider BAR/BOCR or move to a monitored area	Continued security presence<br>Move to a monitored area<br>ED consultant/senior registrar/CMO (UCC) involvement
**Medication**	**Diazepam PO**<br>10–20 mg PO<br><br>Repeat in 2 hours if necessary	**Olanzapine PO/SL**<br>5-10 mg PO/SL<br><br>AND/OR<br><br>**Diazepam PO**<br>10-20 mg PO if not given already<br><br>Repeat in 2 hours if necessary	**Droperidol IM**<br>10 mg IM<br>If remains unsettled in 15 minutes<br><br>**Repeat Droperidol**<br>10 mg IM<br><br>If remains unsettled in a further 15 minutes<br>**Escalate to Behavioural Emergency**	Consider<br>**Midazolam IM/IV***<br>5-10 mg IM<br>OR<br>2.5-5 mg IV<br><br>OR<br><br>**Ketamine IM/IV***<br>4-5 mg/kg IM<br>OR<br>1-2 mg/kg IV

* No IV Medication to be given at UCC's

8.3 Older Adults > 65 years

General Considerations:

- Organic causes are common – consider:
  - Delirium and cause, e.g. sepsis;
  - Dementia/BPSD;
  - Primary psychosis;
  - Intoxication, withdrawal and general side effects of usual medications and other drugs;
  - Pain and toileting requirements;
  - Frailty – vulnerability to complications/side effects;
  - Urinary retention.
- Increased sensitivity to effects and side effects of sedative medications.
- Use the lowest effective dose ("low and slow").
- Oral dosing is preferred.
- Minimise interventions and invasive procedures/investigations (e.g. IDC, IVC).
- Some behaviours may not respond to medication (e.g. Wandering, calling out, impulsivity, falls risk).

If appropriate, all patients should:

- Be nursed in a quiet area.
- Have continuing verbal de-escalation.
- Be provided with food and fluids and toileting access.
- Be considered for an Additional care resource
- If admitted, be prioritised for ward admission

Before considering pharmacological measures:

- Consider de-escalation: low stimulus area, talking down, timeout, distraction, use of relatives if suitable, BAR/BOCR.
- Consider the effect of the environment on behaviour, and expedite disposition with the involvement of other care providers.
- Address fears and concerns.
- Take a history, check drug/alcohol use, allergies, and consider drug interactions and contraindications.
- Check for topically applied medication patches.
- Do a physical examination and appropriate investigations for organic causes.
- Look for features of Parkinsonism.
- Do a thorough neurological examination.
- Do a mental state assessment CAM or 4AT (see *OG-GC2 Delirium Guidelines*).
- Establish a working diagnosis.

Principles of pharmacological management of acute behavioural disturbance in the elderly:

- Atypical anti-psychotics (olanzapine, quetiapine, risperidone) have a lower risk of exacerbating movement disorders than typical anti-psychotics (haloperidol or droperidol).
- Use of more than one anti-psychotic is not recommended.
- Benzodiazepines should be restricted to a single dose or very short-term only.
- Monitor for side effects and complications for both oral and parenteral dosage.
- Maximum doses are provided as a guide. If doses listed below do not achieve an adequate response, obtain senior or specialist clinician advice.
- IV dosings can take 15-30 minutes to peak effect; IM can take up to 60 minutes. Re-dosing in this time-frame increases adverse effects.
- All patients receiving IM or IV medications should be managed in a critical care environment with full monitoring and an ECG.
- Avoid combining parenteral anti-psychotic and benzodiazepine medications.
- When choosing an initial dose, consider age, comorbidities, acute illness, degree of agitation.
- Ensure NOK in contacted for consent before given sedation unless emergency situation.

Risk assessment:

- All patients are subject to the behaviour of concern observations (*Emergency Department Adult Flow Chart – Behaviours of Concern Observations WHAD51.2a*).
- BOC scores > 2 generally require a coder grey and pharmacological management.
- BOC scores of 2 and below should not preclude pharmacological management if other risk factors (e.g. history of violence and disruptive behaviour or worrying trends) are present.

**Patient Behaviour**	**Mild Agitation BOC-score 0**	**Escalating Behaviour BOC-score 1-2**	**Behavioural Crisis BOC-score >2**	**Behavioural Emergency BOC-score >2**
	Anxious and agitated	Becoming more distressed and agitated with a	Severe agitation and distress posing a risk	Persisting aggressive behaviour despite sedation

	But able to be redirected and reassured	possible risk of unintended aggression	to patient and staff safety	
**Aim of Intervention**	Maintain safe environment	Prevent a crisis situation	Restore a safe environment	Maintain staff and patient safety at all times
**Immediate Considerations**	Prefer non-pharmacological strategies	Maximise non-pharmacological strategies<br>Consider BAR/BOCR<br>Low stimulus environment	Code grey – Security present<br>Least restrictive practice<br>Consider BAR/BOCR if appropriate	Continued security presence<br>Move to a monitored area<br>ED consultant/senior registrar/ CMO (UCC) involvement.
**Medications**	Choose ONE of the following:<br><br>**Haloperidol (if no history of Parkinsonism) PO**<br>0.25-0.5 mg PO<br>repeat after 2 hours (max 3 mg/24hours)<br>OR<br>**Quetiapine (preferred in Parkinsonism) PO**<br>12.5-25 mg PO<br>Repeat after 2 hours (max 100 mg/24 hours)<br>OR<br>**Olanzapine PO**<br>2.5 mg PO<br>repeat after 2 hours (max 5 mg /24 hrs)<br>OR<br>**Risperidone (if already on it from RCF) PO**<br>0.5 mg PO<br>repeat after 2 hours (max 2 mg/24 hrs)		Choose ONE of the following:<br><br>**Haloperidol (if no history of Parkinsonism) IM/IV ^***<br>0.5-1 mg IM<br>repeat after 1 hour (max 3 mg/24 hours)<br>OR<br>0.5-1 mg IV<br>repeat after 30 min (max 3 mg/24 hours)<br>OR<br>**Midazolam IM/IV**<br>1-2 mg IM – SINGLE DOSE ONLY<br>(max 2 mg/24 hours)<br>0.5-1 mg IV repeat after 15-30 min<br>(max 2 mg/24 hours)<br>OR<br>**Olanzapine IM**<br>2.5-5 mg IM SINGLE DOSE ONLY<br>(max 5 mg/24 hours)	

^ if given IV needs ECG monitoring for QT prolongation and arrhythmias
* NO IV Medication to be given at UCC's

8.4 General Precautions Applicable to All Patients

8.4.1 List of Medications, Pharmacokinetics and Side Effects

Agent	Peak effect	Half-life	Side effects and interactions
**Benzodiazepines**			
Diazepam	PO: 30-90 minutes	30 hours	Drowsiness, hypotension, respiratory depression<br>Paradoxical agitation and anxiety
Midazolam	IV: 5-15 minutes<br>IM: 15-45 minutes	2 hours	
**Anti-psychotics**			
Olanzapine	PO, SL: 5-8 hrs<br>IM: 15-45 minutes	33 hours	<u>Variable effects with different agents</u>:<br>Hypotension, sedation, bradycardia or tachycardia<br>Extrapyramidal reactions – akathisia, dystonia, tardive dyskinesia, Parkinsonism<br>Neuroleptic malignant syndrome – muscle rigidity, fever, autonomic instability, delirium<br>Prolonged QT with potential for torsades de pointes<br>Seizures (very rarely)
Quetiapine	PO: 1.5 hrs	7 hours	
Risperidone	PO: 1-2 hrs	3 - 19 hours	
Haloperidol	IM, IV:<br>15-30 mins	20 hours	
Droperidol	IM: 5-10 minutes	2 hours	
Ketamine	IM/IV 20 minutes	2.5 hours	Tachycardia, Hypertension, Emergence reaction,

8.4.2 Management of Adverse Effects

Problem	Management (also mandates notification of doctor)
Acute dystonia	Benzatropine IV or IM (oral administration in mild cases possible)<br>Adults - 0.5-2 mg
Respiratory Rate < 8	Give oxygen<br>Consider flumazenil (following administration of benzodiazepines)<br>0.2 mg IV initial dose<br>Then 0.1 mg at 1 minute intervals up to max 1 mg<br>NB: Seizures may be precipitated in benzodiazepine tolerant/dependent patients<br>Consider naloxone if suspicion of opiate use<br>0.1-0.4 mg IV initial dose<br>0.4 mg at 1 minute intervals up to max 1mg<br>Note: Acute opiate withdrawal state may be precipitated in opiate dependent patients

Hypotension	Lie patient flat, tilt bed<br>Ensure monitoring<br>IV fluids
Hyperthermia	Withhold anti-psychotics, check CK
Emergence reaction	Supportive treatment, consider benzodiazepines

8.4.3 Sedation Assessment Tool (SAT)

Score	Responsiveness	Speech
+3	Combative, violent, out of control	Continual loud outbursts
+2	Very anxious and agitated	Loud outbursts
+1	Anxious/restless	Normal/talkative
0	Awake and calm/cooperative	Speaks normally
-1	Asleep but rouses if name is called	Slurring or prominent slowing
-2	Responds to physical stimulation	Few recognisable words
-3	No response to stimulation	Nil

9. Document History

Number of previous revisions: 1

Previous issue dates: February 2020

10. References

1. MIMS
2. AMH Australian Medicines Handbook
3. AMH Aged Care companion
4. AMH Children's Dosing Companion
5. Therapeutic Guidelines: Psychotropic, Version 7, Therapeutic Guidelines Limited
6. Royal Childrens Hospital Melbourne Clinical Practice Guidelines, Acute Behavioural disturbance
7. Taylor,D; Paton, C & Kapur, S. The Maudsley Prescribing Guidelines in Psychiatry – 13th edition. (2018)
8. Delirium Clinical Care Standard at https://www.safetyandquality.gov.au/our-work/clinical-care-standards/delirium-clinical-care-standard/
9. Australian Injectable Drug Handbook (AIDH)
10. Australian Pharmaceutical Formulary (APF)
11. Micromedex

11. Sponsor

Director of Emergency Medicine Program

12. Authorisation Authority

Clinical Services Director of Emergency Medicine and Access

Appendix 1

MANAGEMENT OF ACUTE BEHAVIOURAL DISTURBANCE IN THE EMERGENCY DEPARTMENT

Children <16years or < 50kg

RISK ASSESSMENT<br>What is the situation?				
**Patient Behaviour**	**Mild Agitation**<br><br>Anxious and agitated<br>But able to be redirected and reassured	**Escalating Behaviour**<br><br>Becoming more agitated with possible risk of loss of self-control and aggression	**Behavioural Crisis**<br><br>Severe agitation and distress posing risk to patient and staff safety	**Behavioural Emergency**<br><br>Persisting aggressive behaviour despite sedation
**Aim of Intervention**	Maintain safe environment	Prevent a crisis situation	Restore a safe environment	Maintain staff and patient safety at all times
**Immediate Considerations**	Introduce non-pharmacological strategies, involve family member if appropriate	Maximise non-pharmacological strategies<br>Consider BAR/BOCR<br>Low stimulus environment	Code grey – Security present<br>Least restrictive practice<br>Consider BAR/BOCR if appropriate	Continued security presence<br>Move to a monitored area<br>ED consultant/senior registrar/ UCC CMO involvement
**Medication**	In a child with known behavioural disorder consider giving additional dose of usual medication<br>**Olanzapine wafer SL**<br><40kg 2.5-5 mg SL (max 10 mg daily)<br>>40kg 5-10 mg SL (max 20 mg daily)<br>or<br>**Diazepam PO (available in liquid form)**<br>0.2-0.4 mg/kg PO<br>Max 10 mg		**Droperidol IM**<br>0.1-0.2 mg/kg<br>(max 10 mg)<br>Or<br>**Olanzapine IM**<br><40kg 5 mg<br>>40kg 10mg<br>Repeat in 15 min – if not settling **escalate to Behavioural Emergency**	**Ketamine IM/IV***<br>4 mg/kg IM<br>(max 400 mg)<br>OR<br>1 mg/kg IV<br>(max100 mg)<br>Or<br>**Midazolam IM/IV***<br>0.1-0.2 mg/kg<br>IM or IV<br>(max 20 mg/24 hours)<br>* No IV Medication to be given at UCC's

Appendix 2

MANAGEMENT OF ACUTE BEHAVIOURAL DISTURBANCE IN THE EMERGENCY DEPARTMENT
Adults < 65years

**RISK ASSESSMENT**<br>**What is the situation?**				
**Patient Behaviour**	**Mild Agitation**<br>**BOC-score 0**<br><br>Anxious, agitated<br>But able to be redirected and reassured	**Escalating Behaviour**<br>**BOC-score 1-2**<br><br>Becoming more agitated with possible risk of loss of self-control and aggression	**Behavioural Crisis**<br>**BOC-score >2**<br><br>Aggressive behaviour is overt, imminent safety threat	**Behavioural Emergency**<br>**BOC-score >2**<br><br>Persisting aggressive behaviour despite sedation
**Aim of Intervention**	Maintain safe environment	Prevent a crisis situation	Restore a safe environment	Maintain staff and patient safety at all times
**Immediate considerations**	Consider BAR/BOCR<br>Low stimulus environment	Consider BAR/BOCR<br>Low stimulus environment	Code grey<br>Consider BAR/BOCR<br>or move to a monitored area	Continued security presence<br>Move to a monitored area<br>ED consultant/senior registrar/ UCC CMO involvement
**Medication**	**Diazepam PO**<br>10-20 mg PO<br><br>Repeat in 2 hours if necessary	**Olanzapine PO/SL**<br>5-10 mg PO/SL<br><br>And/OR<br><br>**Diazepam PO**<br>10-20 mg PO if not given already<br><br>Repeat in 2 hours if necessary	**Droperidol IM**<br>10 mg IM<br>If remains unsettled in 15 minutes<br>**Repeat Droperidol IM**<br>10 mg IM<br><br>If remains unsettled in a further 15 minutes<br><br>**Escalate to Behavioural Emergency**	consider<br>**Midazolam IM/IV***<br>5-10 mg IM<br>OR<br>2.5-5 mg IV<br><br>OR<br><br>**Ketamine IM/IV***<br>4-5 mg/kg IM<br>OR<br>1-2 mg/kg IV<br>* No IV Medication to be given at UCC's

Appendix 3

MANAGEMENT OF ACUTE BEHAVIOURAL DISTURBANCE IN THE EMERGENCY DEPARTMENT
Older Adults >65years

**RISK ASSESSMENT<br>What is the situation?**				
**Patient Behaviour**	**Mild Agitation**<br>**BOC-score 0**<br>Anxious and agitated<br>But able to be redirected and reassured	**Escalating Behaviour**<br>**BOC-score 1-2**<br><br>Becoming more distressed and agitated with possible risk of unintended aggression	**Behavioural Crisis**<br>**BOC-score >2**<br><br>Severe agitation and distress posing risk to patient and staff safety	**Behavioural Emergency**<br>**BOC-score >2**<br><br>Persisting aggressive behaviour despite sedation
**Aim of Intervention**	Maintain safe environment	Prevent a crisis situation	Restore a safe environment	Maintain staff and patient safety at all times
**Immediate Considerations**	Prefer non pharmacological strategies	Maximise non pharmacological strategies<br>Consider BAR/BOCR<br>Low stimulus environment	Code grey – Security present<br>Least restrictive practice<br>Consider BAR/BOCR if appropriate	Continued security presence<br>Move to monitored area<br>ED consultant/senior registrar/ UCC CMO involvement
**Medications**	Choose one of:<br>**Haloperidol (if no history of Parkinsonism) PO**<br>0.25-0.5mg PO<br>repeat after 2 hours (max of 3mg/24hours)<br>OR<br>**Quetiapine (preferred in Parkinsonism) PO**<br>12.5-25 mg PO<br>Repeat after 2 hours (max 100 mg/24 hours)<br>OR<br>**Olanzapine PO**<br>2.5mg PO – repeat after 2 hours (max 5 mg /24 hours)<br>OR<br>**Risperidone (if already on it from RCF) PO**<br>0.5 mg PO - repeat after 2 hours (max 2 mg/24 hours)		Choose one of:<br>**Haloperidol (if no history of Parkinsons) IM/IV***<br>0.5-1mg IM - repeat after 1 hour (max of 3mg/24 hours)<br>OR<br>0.5-1mg IV - repeat after 30 minutes<br>(max 3 mg/24 hours)<br>OR<br>**Midazolam – IM/IV* - Single dose only**<br>1-2mg IM (max 2 mg/24 hours)<br>0.5-1mg IV - repeat after 15-30 minutes<br>(max 2mg/24hours)<br>OR<br>**Olanzapine – IM - Single dose only**<br>2.5-5mg IM (max 5 mg/24 hours)<br>* No IV Medication to be given at UCC's	

Appendix 4: Behaviours of Concern (BOC) Chart

GLASGOW COMA SCORE (if total score fall by 2 or more, inform MO)																							
			ADULT TIME:																				
LEVEL OF CONSCIOUSNESS	Eyes open	4	Spontaneously																				
		3	Speech																				
		2	Pain																				
		1	None																				
	Best verbal response	5	Orientate																				
		4	Confused																				
		3	Inappropriate																				
		2	Incoherent																				
		1	None																				
	Best motor response	6	Obey command																				
		5	Localise pain																				
		4	Withdraws																				
		3	Abn flexion																				
		2	Abn extension																				
		1	None																				
TOTAL COMA SCORE																							
PUPILS	Right eye		Size																				
			Reaction																				
	Left eye		Size																				
			Reaction																				
ARMS			Normal power																				
			Mild weakness																				
			Mod. Weakness																				
			Severe weakness																				
			Spasticity																				
			None																				
LEGS			Normal power																				
			Mild weakness																				
			Mod. Weakness																				
			Severe weakness																				
			Spasticity																				
			None																				

BEHAVIOURS OF CONCERN OBSERVATIONS (On arrival, half-hourly until MO, then hourly as for other observations)																				
Score of 0 or 1 for behaviour TIME:																				
**CONFUSED** Obviously confused or disorientated																				
**IRRITABLE** Easily annoyed or angered, intolerant																				
**BOISTEROUS** Overtly noisy, shouts, slams doors																				
**PHYSICAL THREATS** Threatening or intimidating stance or gestures																				
**VERBAL THREATS** Threatening or intimidating language																				
**ATTACKING OBJECTS** Kicking, hitting, throwing, damaging objects																				
TOTAL SCORE (Maximum of 6)																				

MANAGEMENT MATRIX	SCORE = 0	SCORE = 1-2	SCORE >2
	**Risk of Violence**: SMALL<br>•No required interventions	**Risk of Violence**: MODERATE<br>•Consider preventative measures	**Risk of Violence**: VERY HIGH<br>•Ensure safety<br>•Plan for potential deterioration
GENERAL	•Continue regular observations<br>•Search EDIS (and Bossnet) for alerts for behaviours of concern	•Ensure personal safety<br>•Call for help if required<br>•Avoid/manage triggers as able<br>•Consider Code Grey if appropriate	**As for moderate PLUS**<br>•CALL CODE GREY<br>•Notify admitting team of clinical risk<br>•Document incident including triggers and
NURSING	•Notify NIC if previous alerts for behaviours of concern<br>•NIC: Notify security	•Notify NIC & ED Medical Officer<br>•Request PRN medications to be charted by ED Medical Officer if not already<br>•Offer oral medications	•Attempt verbal deescalation if safe<br>•Offer oral medications if appropriate
MEDICAL (ED)	•Chart PRN medications for ALL mental health patients	•Chart PRN medications for ALL mental health patients<br>•Assist nursing with preventing escalation	•Attempt verbal deescalation if safe<br>•Offer oral medications if appropriate<br>•Consider IV/IM sedation if unsuccessful<br>•Follow Rapid Sedation Guideline
SECURITY	•Be aware of all patients with alerts for behaviours of concern	•Increase presence<br>•Liaise with beside nurse regularly	•Attend Code Grey & provide assistance as<br>•Provide mechanical restraints as requested<br>•Document incident

Appendix 5: HEADSSS Assessment (Psychosocial Screening)

RCH clinical practice guidelines: Engaging with and assessing the adolescent patient

**Home:**	Including who, where, recent moves, relationships, violence.
**Education & Employment:**	Including where, attendance, year, performance, relationships, supports, recent moves, bullying, disciplinary actions, future plans, and work details
**Eating:**	Including weight (heaviest, lightest, recent changes), dieting, exercise and menstrual history.
**Activities:**	outside of school, including sport, organised groups, clubs, parties, TV/ computer use
**Drugs and Alcohol:**	Including cigarettes, alcohol and illicit drug use by friends, family and the patient. Enquire into patterns & frequency of use & about any regrets from using these substances.<br>Also ask about how use is financed and about negative consequences.
**Sexuality:**	Including close relationships, sexual experiences, number of partners (total and in the last 3 months), gender of sexual partners (don't assume sexual preferences), uncomfortable situations/ sexual abuse, risk of pregnancy and previous pregnancies (relevant to males as well as females), contraception, condoms and STIs.
**Suicide, Depression & Self-harm:**	Presence and frequency feeling down or sad as well as current feelings eg. "How do you feel in yourself at the moment on a scale of 1 to 10?" Actions when down, supports. Self-harm- thoughts and actions. Suicide risk- thoughts, attempts, plans, means and hopes for future.
**Safety from injury &Violence:**	Including serious injuries, use of safety gear for sports and seatbelts for cars, riding with an intoxicated driver and exposure to violence at school and in neighbourhood.<br>For high risk youths ask about carrying or use of weapons and other criminal behaviours/ incarceration of youth or family/ friends.

Appendix 1 (if applicable)
